# Prospective study on smoking and postoperative complications in breast reconstruction surgery

**DOI:** 10.6026/973206300213071

**Published:** 2025-09-30

**Authors:** Shreyanshi Satyambhai Vijakar, Vishal Mruthyunjaya Kalmani, Mansoor Thekkinithethil, Loisen Nathan Daniel, Sailesh I.S. Kumar

**Affiliations:** 1 Department of Pathology, Narendra Modi medical college, Ahmedabad, Gujarat, India; 2 Department of Emergency Medicine, Barking, Havering and Redbridge University Hospitals NHS Trust, Essex, United Kingdom; 3 Department of Trauma and Orthopedics, Junior Clinical Fellow Trauma and Orthopaedics, Swansea Bay University Health Board JCF in T & O, United Kingdom; 4 Department of Emergency Medicine/intensive care/general medicine, Best Hospital, Tamil Nadu, India; 5 Department of Surgery, Madras Medical College, Tamil Nadu, India

**Keywords:** Smoking, breast reconstruction, postoperative complications, wound healing, flap necrosis, prospective study

## Abstract

The impact of smoking on postoperative complications in 125 women undergoing breast reconstruction surgery is of interest. Hence, patients were stratified into current smokers, former smokers and non-smokers and followed for 30 days postoperatively. Current smokers had significantly higher rates of wound dehiscence, infection and flap necrosis compared to non-smokers. Former smokers showed intermediate complication rates. Thus, we show the critical need for smoking cessation strategies prior to reconstructive breast surgery.

## Background:

Smoking is a cause of many postoperative complications, including delayed wound healing, tissue necrosis and reconstructive flap loss. However, there is a paucity of evidence-based guidelines for smoking cessation in patients undergoing implant-based breast surgery [1]. Breast reconstruction following mastectomy is a critical component of comprehensive breast cancer care, offering physical restoration and psychological benefits [2]. However, the procedure is not without risk and postoperative complications can significantly impact aesthetic outcomes, patient satisfaction and long-term recovery [3]. Among the modifiable risk factors, cigarette smoking has consistently been associated with impaired wound healing, increased risk of infection and compromised flap viability in various surgical settings [4]. Nicotine and other tobacco-derived toxins contribute to vasoconstriction, impaired oxygen delivery, reduced collagen synthesis and increased oxidative stress all of which negatively affect tissue repair [5]. Despite these well-known physiological effects, many patients' undergoing breast reconstruction are not adequately counseled or supported in smoking cessation prior to surgery [6]. Moreover, few prospective studies have systematically evaluated the differential risks between current smokers, former smokers and non-smokers in this specific surgical population [7]. Therefore, it is of ineterest to investigate the relationship between smoking status and the incidence of postoperative complications in women undergoing breast reconstruction.

## Materials and Methods:

This prospective observational study was conducted over a 12-month period in the Department of Plastic and Reconstructive Surgery at a tertiary care academic hospital. A total of 125 female patients undergoing breast reconstruction surgery following mastectomy for breast cancer were enrolled after obtaining informed consent. Both immediate and delayed reconstructions were included, encompassing autologous flap procedures (such as DIEP and TRAM flaps) and implant-based reconstructions. Patients with severe systemic illness, prior radiotherapy to the chest wall, active infections, or poorly controlled diabetes were excluded to eliminate confounding influences on wound healing. Smoking history was obtained during preoperative evaluation and verified through patient interviews and medical records. Participants were categorized into three groups: current smokers (those actively smoking within 30 days of surgery), former smokers (abstinent for at least 30 days) and non-smokers (never smoked or smoked fewer than 100 cigarettes lifetime). Data collected included age, body mass index (BMI), comorbidities (hypertension, diabetes), alcohol use, type and timing of reconstruction, duration and intensity of smoking (pack-years) and medication history. All patients underwent standardized surgical techniques and perioperative care protocols. Postoperative follow-up was conducted for 30 days to document wound-related and systemic complications. Primary outcomes included surgical site infection, wound dehiscence, hematoma, seroma, flap necrosis, delayed wound healing and need for reoperation. Data analysis involved comparison of complication rates across the three smoking groups using chi-square tests for categorical variables and ANOVA for continuous variables. Logistic regression was used to assess the independent impact of smoking status on complications, controlling for age, BMI, comorbidities and type of reconstruction. A p-value <0.05 was considered statistically significant.

## Results:

This prospective study demonstrated a clear association between smoking status and increased postoperative complications in breast reconstruction surgery. Current smokers had significantly higher rates of wound-related issues-including infection, dehiscence and flap necrosis-compared to non-smokers and former smokers. Former smokers showed intermediate complication rates, reinforcing the benefit of preoperative cessation. Table 1 (see PDF) shows the patient cohort was predominantly middle-aged, with a majority undergoing implant-based reconstruction. Current smokers had higher BMI and more comorbidity. Table 2 (see PDF) shows current smokers experienced the highest complication rate overall, especially for infections and delayed healing. Table 3 (see PDF) shows wound infection and delayed healing was significantly more frequent in autologous reconstructions among current smokers. Table 4 (see PDF) shows pack-years correlated positively with complication rates among current and former smokers. Table 5 (see PDF) shows Logistic regression showed smoking status as an independent predictor of postoperative complications. Table 6 (see PDF) shows most complications among smokers occurred within the first 10 days post-surgery. Table 7 (see PDF) shows the reoperation rate was significantly higher among smokers, mainly for debridement and flap salvage. Table 8 (see PDF) shows current smokers had significantly longer hospital stays compared to non-smokers, driven primarily by infection and wound-related delays. Table 9 (see PDF) shows patients who developed complications reported significantly lower satisfaction scores postoperatively, particularly among smokers. Table 10 (see PDF) shows a significant proportion of smoker's required delayed initiation of adjuvant cancer therapy due to wound-related issues.

## Discussion:

This prospective study highlights the significant impact of smoking on postoperative outcomes in breast reconstruction surgery. Our findings clearly demonstrate that current smokers are at markedly increased risk for complications, including surgical site infection, delayed wound healing, flap necrosis and the need for reoperation [8]. Former smokers also experienced elevated complication rates, though substantially lower than current smokers, suggesting a partial but not complete reversal of smoking-related surgical risk with cessation [9]. Nicotine-induced vasoconstriction, carbon monoxide-mediated hypoxia and impaired fibroblast activity are well-established mechanisms by which smoking interferes with wound healing [10]. These pathophysiologic effects were evident in our cohort, particularly in autologous flap reconstructions, where perfusion and microvascular integrity are critical. Smokers undergoing flap-based procedures experienced high rates of necrosis and reoperation, reinforcing the importance of adequate perfusion in high-risk surgical environments. The correlation between pack-years and complication rates provides additional evidence for a dose-dependent effect of smoking on wound healing [11]. Patients with greater than 10 pack-years had the highest overall complication rates, suggesting that both current behavior and cumulative exposure are clinically relevant. This observation supports early and aggressive preoperative counseling, especially for long-term smokers. Interestingly, most complications in current smokers manifested within the first week postoperatively, indicating that acute surgical stress in a compromised vascular environment may precipitate early wound failure [12]. Reoperation was largely driven by flap necrosis and infection, which not only jeopardize aesthetic outcomes but also prolong hospitalization and increase healthcare costs. The logistic regression analysis confirmed smoking as an independent predictor of postoperative morbidity, even after adjusting for other confounding factors such as BMI, diabetes and reconstruction type [13]. This finding underscores the necessity of incorporating smoking status into preoperative risk stratification models and reinforces the role of multidisciplinary perioperative interventions [14]. Our study also highlights a window of opportunity in former smokers, who had notably fewer complications than active smokers. This supports existing evidence that even short-term cessation before surgery (30 days or more) can lead to improved surgical outcomes. However, their intermediate complication rates also suggest that the physiological effects of long-term tobacco exposure are not fully reversible in the short term. In clinical practice, these results advocate for stronger preoperative protocols emphasizing smoking cessation, ideally initiated well in advance of planned breast reconstruction. Patients should be informed of the heightened risks associated with smoking and institutions should facilitate access to cessation resources as a routine part of surgical planning [15]. In summary, smoking is a potent, modifiable risk factor for adverse outcomes in breast reconstruction surgery. The data strongly support integrating tobacco cessation as a core element of preoperative care to enhance healing, reduce complications and improve reconstructive success.

## Conclusion:

Smoking significantly increases the risk of postoperative complications in breast reconstruction, including infection, wound dehiscence and flap necrosis. Former smokers show reduced but still elevated risk compared to non-smokers. Preoperative smoking cessation is essential to improve surgical outcomes and recovery.
